# Risk prediction for breast Cancer in Han Chinese women based on a cause-specific Hazard model

**DOI:** 10.1186/s12885-019-5321-1

**Published:** 2019-02-07

**Authors:** Lu Wang, Liyuan Liu, Zhen Lou, Lijie Ding, Hui Guan, Fei Wang, Lixiang Yu, Yujuan Xiang, Fei Zhou, Fuzhong Xue, Zhigang Yu

**Affiliations:** 1 Department of Biostatistics, School of Public Health, Shandong University, 44 Wen Hua Xi Road, Jinan, 250012 China; 2 Department of Breast Surgery, the Second Hospital of Shandong University, Jinan, 250033 China; 3 Institute of Translational Medicine of Breast Disease Prevention and Treatment, Shandong University, Jinan, 250033 China; 4 Shandong University of Finance and Economics, Jinan, 250014 China; 5 Division of Health Management, School of Sport Social Science, Shandong Sport University, Jinan, 250102 China; 6 Department of Radiotherapy, Fourth People’s Hospital of Jinan, No 50 Shifan Road, Jinan, 250031 Shangdong China; 7 Suzhou Institute of Shandong University, Suzhou, 215123 China

**Keywords:** Breast neoplasms, Population at risk, Risk assessment

## Abstract

**Background:**

Considering the lack of efficient breast cancer prediction models suitable for general population screening in China. We aimed to develop a risk prediction model to identify high-risk populations, to help with primary prevention of breast cancer among Han Chinese women.

**Methods:**

A cause-specific competing risk model was used to develop the Han Chinese Breast Cancer Prediction model. Data from the Shandong Case-Control Study (328 cases and 656 controls) and Taixing Prospective Cohort Study (13,176 participants) were used to develop and validate the model. The expected/observed (E/O) ratio and C-statistic were calculated to evaluate calibration and discriminative accuracy of the model, respectively.

**Results:**

Compared with the reference level, the relative risks (RRs) for highest level of number of abortions, age at first live birth, history of benign breast disease, body mass index (BMI), family history of breast cancer, and life satisfaction scores were 6.3, 3.6, 4.3, 1.9, 3.3, 2.4, respectively. The model showed good calibration and discriminatory accuracy with an E/O ratio of 1.03 and C-statistic of 0.64.

**Conclusions:**

We developed a risk prediction model including fertility status and relevant disease history, as well as other modifiable risk factors. The model demonstrated good calibration and discrimination ability.

**Electronic supplementary material:**

The online version of this article (10.1186/s12885-019-5321-1) contains supplementary material, which is available to authorized users.

## Background

Breast cancer is one of the most prevalent malignancies among women worldwide [1]. Although the incidence of breast cancer is low compared with Western countries, China is currently experiencing increasing trends in both breast cancer incidence and mortality [2]. However, the mammography screening participation rate is only 21.7% in China, far lower than in Western countries [3]. Considering the limited medical resources, especially in rural areas of China, a risk prediction model that is suitable for general population screening is urgently needed.

Competing risks are said to be present when an individual is at risk for more than one mutually exclusive event, such as death from a different cause, and the occurrence of one of these competing events will prevent the event of interest from ever happening. A cause-specific hazard model considers competing risks and therefore has better performance than the Cox model when used for disease risk prediction. Gail proposed a method to estimate individual probabilities of developing breast cancer, based on a cause-specific hazard model [4]. Several risk prediction models have been developed in Western countries; the two most widely used are the Gail cause-specific hazard model with traditional risk factors as predictors [4], and the International Breast Cancer Intervention Study (IBIS)model, which includes genetic markers [5]. The Gail II model was developed based on the Gail model and provides a feasible web-based instrument [6]; however, that model was first developed in a Caucasian ethnic population so the effects maybe uncertain when directly applied to Han Chinese women [4, 6–11]. Moreover, because biopsies are not widely used in China (especially in rural areas), information on the number of previous breast biopsies is not readily available for most Chinese women. Although other prediction models include genetic risk prediction models such as the IBIS model [12], their higher cost may lead to limited application considering the large population in China. In addition, the most important genetic factor of breast cancer, the *BRCA* gene mutation rate, is lower in the Chinese population than those in Western countries [13]. The aim of our study was to develop a breast cancer risk prediction model using non-laboratory indicators in a population of Han Chinese women, and to convert the model into a simple tool that can be conveniently used in clinical and public health practice.

## Methods

### Study population

All participants were selected from the study population of the project “Ministry-affiliated Hospital Key Project of the Ministry of Health of the People’s Republic of China (No. 07090122)”. We conducted a population-based case-control study in Shandong Province to develop the prediction model, which was then validated in the Taixing Prospective Cohort Study. The study protocol was approved by Shandong University and informed consent was obtained from all study participants.

In the population-based Shandong Case-Control Study, which was conducted from 2008 to 2012, the target population included 25- to 70-year-old women of Han ethnicity, with over 2 years of local residence and at least 6 months of local residence at the time of the survey. Breast cancer patients were from the department of breast surgery at the Second Hospital of Shandong University. All patients were newly diagnosed with breast cancer. Each eligible patient who had been pathologically diagnosed with breast cancer was matched with two healthy controls according to age (± 2 years) and location (neighbor or co-worker in the same region). Finally, 328 cases and 656 controls were included in the Shandong Case-Control Study. Details of the questionnaire used in this study are described elsewhere [2, 14].

**Fig. 1:**
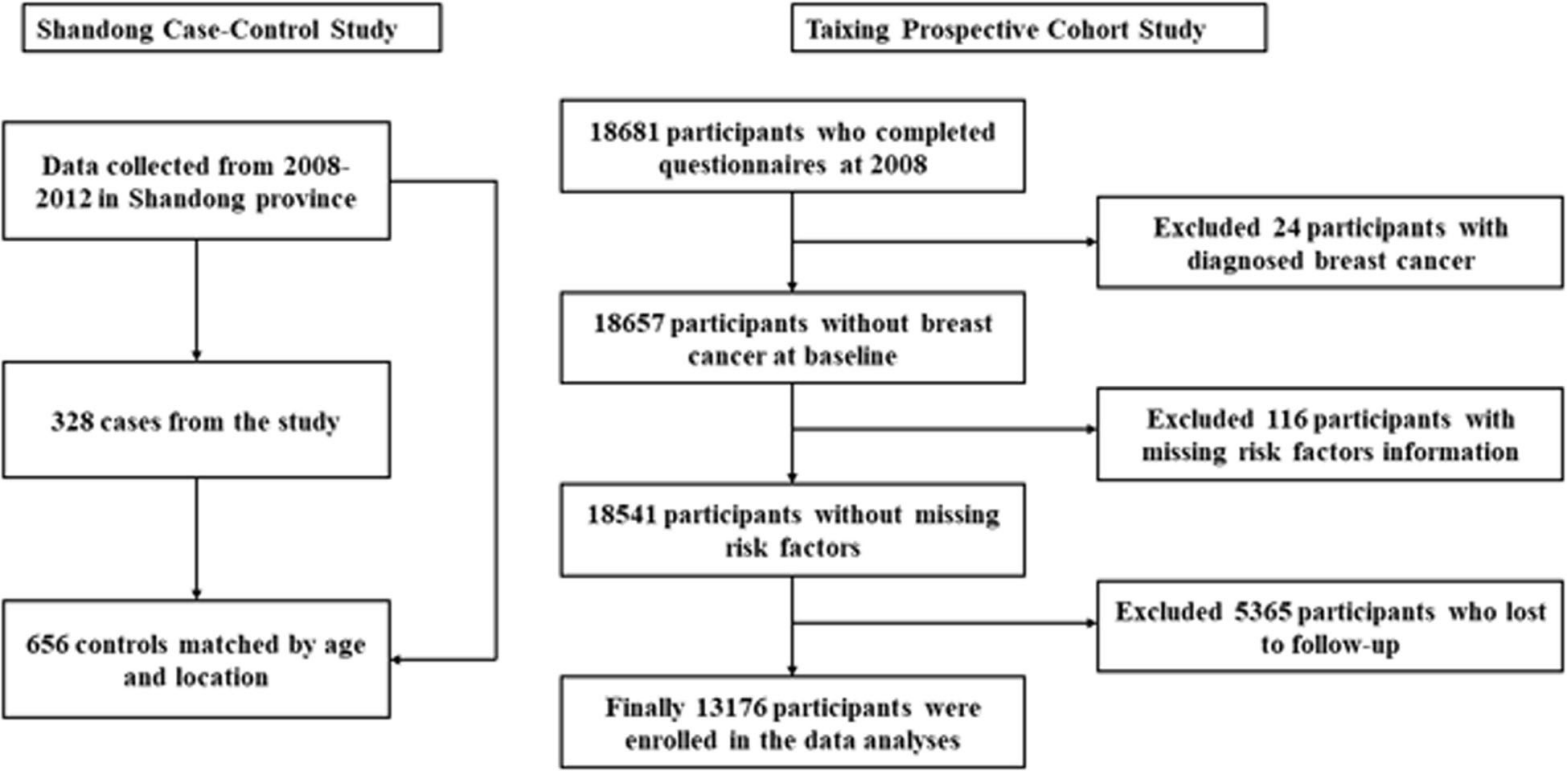
Flow chart of the enrollment in Shandong Case Control Study and Taixing Prospective Cohort Study

In the Taixing Prospective Cohort Study, 18,681 participants who completed a questionnaire in 2008 were included as the baseline population, and outcomes were collected after 7 years of follow-up. Participants who have not been diagnosed with breast cancer at baseline were selected for the study (*n* = 18,657), after excluding participants with missing data of risk factors (*n* = 116). Among 18,541 eligible participants, 5365 participants were lost to follow-up. The rate of lost to follow-up was 28.9% (owing to adjustment of the administrative jurisdiction, one township was excluded from Taixing City when collecting follow-up information). Finally, 13,176 individuals were enrolled. New incident cases of breast cancer were identified from local cancer registries and via active follow-up telephone and face-to-face interviews. The flow chart of the enrollment in these two studies was shown in Fig. 1.

### Measurements and definition of risk factors

A self-designed structured questionnaire that included demographic characteristics, female physiological and reproductive factors, medical and family history, dietary habits, lifestyle habits, and breast-cancer-related knowledge was used to obtain data [2]. Data were collected via in-person interviews. Body mass index (BMI) was calculated as weight in kilograms divided by height in meters squared; BMI was divided into < 24, 24–27.9, ≥28 corresponding to normal weight, overweight, and obesity [15]. Height was measured using a meter rule with a precision of 0.1 cm, and weight was measured using an electronic weight scale with an accuracy of 0.1 kg. Life satisfaction scores (with respect to housing, income, health, marriage, medical care, and neighbors) were measured using 1/2/3/4/5 scale where 1 is very satisfied and 5 is very unsatisfied. A total point of 30 was divided into two levels by the mean value 13, people with scores lower than 13 were defined as satisfied and scores ≥13 were defined as unsatisfied. A positive breast cancer family history was defined as any first-, second-, or third-degree relative with a diagnosis of breast cancer. Information on the number of abortions, age at first live birth, and history of benign breast disease were also collected using the questionnaire. All of the above variables have been reported as potential risk factors of developing breast cancer. The coding for the six risk factors is shown in Table 1.

**Table 1:** Characteristics of the Shandong Case-Control Study and baseline characteristics of the Taixing Prospective Cohort Study

Variables		Code	Shandong Case Control Study			Taixing Prospective Cohort Study		
			case (*n* = 328)	control(*n* = 656)	*P*-value	case(*n* = 34)	control(*n* = 13,142)	*P*-value
Age	Year		49.65 ± 9.26	49.65 ± 9.26	0.992	47.06 ± 9.45	46.78 ± 11.46	0.888
Number of abortions	0	0	137 (41.77)	460 (70.12)		23 (67.65)	9612 (73.14)	
	1–2	1	164 (50.00)	177 (26.98)	<0.001	10 (29.41)	3210 (24.43)	0.771
	≥3	2	27 (8.23)	19 (2.90)		1 (2.94)	320 (2.43)	
Age at first live birth	< 25	0	128 (39.02)	378 (57.62)		23 (67.65)	9853 (74.97)	
	25–29	1	175 (53.35)	253 (38.57)	<0.001	11 (32.35)	3138 (23.88)	0.438
	≥30	2	25 (7.62)	25 (3.81)		0 (0.00)	151 (1.15)	
Benign breast disease history	Yes	1	18 (5.49)	7 (1.07)	<0.001	0 (0.00)	46 (0.35)	1.000
BMI	< 24	0	143 (43.60)	347 (52.90)		17 (50.00)	9039 (68.78)	
	24–27.9	1	130 (39.63)	233 (35.52)	0.002	14 (41.18)	3690 (28.08)	0.026
	≥28	2	55 (16.77)	76 (11.59)		3 (8.82)	413 (3.14)	
Breast cancer family history	Yes	1	19 (5.79)	9 (1.37)	< 0.001	2 (5.88)	90 (0.68)	0.009
Life satisfaction scores	≥13	1	188 (57.32)	229 (34.91)	<0.001	22 (64.71)	6907 (52.56)	0.213

*Data are mean ± SD and frequency (percent) for numeric and discrete variables

### Statistical methods

To project individual probabilities (absolute risks) of developing breast cancer, we applied the cause-specific hazard model approach [4], according to the following steps.

(1) We estimated the relative risks (RRs) and attributable risk (AR) in the Shandong Case-Control Study.

To estimate RRs, odds ratios (ORs) and the corresponding 95% confidence intervals (CIs) were calculated in conditional logistic regression for matched data using the above variables and coding, as described in Table 1. Variable selection for inclusion in the final model was based on Wald tests for individual parameters as well as information on previously established risk factors. To estimate the AR, we applied Bruzzi’s method [16]. The AR was estimated using the Taixing cohort and was applied to Taixing City only.

(2) We calculated age-specific baseline hazards for breast cancer (based on the breast cancer incidence rate of the Cancer Surveillance System of the Taixing Centers for Disease Control) and for competing events (based on non-breast cancer mortality from the Death Surveillance System of the Taixing Centers for Disease Control).

Twelve age groups were defined (ranges 25–29, 30–34, 35–39, 40–44, 45–49, 50–54, 55–59, 60–64, 65–69, 70–74, 75–79, 80–84 years). The baseline hazard was defined as the hazard rate for each individual whose risk factors were at the lowest risk level. The baseline hazards of breast cancer were estimated by multiplying the age-specific breast cancer incidence rates by (1 – estimated population AR).

(3) We combined baseline hazards (for breast cancer and competing events) and RRs to estimate probabilities in the developed Han Chinese Breast Cancer Prediction (HCBCP) model. The absolute risks were calculated according to initial age, follow-up duration, and initial RRs in the HCBCP model. A simple computing method for individualized risk assessment was performed [17].

(4) Finally, the Taixing Prospective Cohort Study was used to validate the HCBCP model. We used E/O ratios (which are defined as the observed divided by the expected number, with a number close to 1 showing good model fit) to assess model calibration. The C-statistic, which is the probability that a randomly chosen positive instance will rank higher than a randomly chosen negative one, was used to evaluate the model’s discriminatory ability.

SAS version 9.4 (SAS Institute Inc., Cary, NC, USA) was used to perform data cleaning and to calculate RRs. We used R (The R Project for Statistical Computing, Vienna, Austria) to develop the HCBCP model; the corresponding code is provided in Additional file 1.

## Results

### Relative and attributable risks in the Shandong case-control study

Table 1 shows the characteristics of the Shandong Case-Control Study and baseline characteristics of the Taixing Prospective Cohort Study by breast cancer. The average age of participants in these studies was 49.6 and 49.65 years for those with and without breast cancer, respectively, in the Shandong Case-Control Study, and 47.06 and 46.78 years for those with and without breast cancer, respectively, in the Taixing Prospective Cohort Study.

RRs were estimated using multivariate conditional logistic regression. Risk factors that were significant in univariate ordered logistic regression (see Additional file 2: Table S1) and multivariable logistic regression were included in the final model. Diabetes was a risk factor in univariate logistic regression but was not significant after multivariate-adjustment; therefore, it was not included in the final risk model. Table 2 summarizes the RR of each risk factor. Compared with the reference level, the RRs (95% CI) for a highest level of number of abortions, age at first live birth, benign breast disease history, BMI, family history of breast cancer, and life satisfaction score were 6.313 (4.792, 8.316), 3.589 (2.747, 4.690), 4.255 (1.613, 11.229), 1.882 (1.503, 2.356), 3.250 (1.339, 7.890), and 2.424 (1.777, 3.308), respectively. For breast cancer family history, in about 8.9% of women with a positive family history was contributed from third-degree relative; the prevalence of breast cancer among third-degree relatives was 62/100,000. RRs for each participant were obtained by multiplying each risk factor’s relative risk. The estimated AR was 0.78.

**Table 2:** Relative risks estimated using multivariate logistic regression for the Shandong Case-Control Study

Variables		Code	RR	95% *CI*		*P*-value
Number of abortions	0	0	1.000	–		
	1–2	1	2.512	1.907	3.310	<0.001
	≥3	2	6.313	4.792	8.316	
Age at first live birth	< 25	0	1.000	–		<0.001
	25–29	1	1.895	1.45	2.475	
	≥30	2	3.589	2.747	4.690	
Benign breast disease history	No	0	1.000	–		0.003
	Yes	1	4.255	1.613	11.229	
BMI	< 24	0	1.000	–		0.006
	24–27.9	1	1.372	1.096	1.717	
	≥28	2	1.882	1.503	2.356	
Breast cancer family history	No	0	1.000	–		0.009
	Yes	1	3.250	1.339	7.890	
Life satisfaction scores	< 13	0	1.000	–		< 0.001
	≥13	1	2.424	1.777	3.308	

### Individualized absolute risk projections in Taixing prospective cohort study

The age-specific breast cancer incidence rates and non-breast cancer mortality rates in Taixing were shown in Additional file 3: Table S2. Table 3 gave 5 to 30 years absolute risk probabilities predicted using the HCBCP model, based on different initial ages and RRs.

**Table 3:** Projected absolute risk (%) of breast cancer by relative risks, initial age, and follow-up years

Initial age,y	follow-up years	Projected absolute risk, %					
		Relative risk					
		1	5	10	15	20	25
25	10	0.01	0.03	0.06	0.09	0.12	0.15
	20	0.07	0.36	0.71	1.06	1.42	1.77
	30	0.18	0.88	1.74	2.60	3.46	4.30
30	5	0.01	0.03	0.06	0.09	0.12	0.15
	10	0.01	0.06	0.11	0.17	0.22	0.28
	20	0.14	0.69	1.37	2.04	2.71	3.38
	30	0.22	1.09	2.16	3.22	4.27	5.31
40	5	0.06	0.30	0.60	0.91	1.20	1.50
	10	0.13	0.63	1.26	1.89	2.51	3.13
	20	0.21	1.04	2.06	3.08	4.08	5.08
	30	0.28	1.39	2.76	4.10	5.43	6.74
50	5	0.04	0.20	0.40	0.60	0.80	0.99
	10	0.08	0.42	0.83	1.24	1.65	2.06
	20	0.16	0.78	1.55	2.31	3.07	3.82
	30	0.20	1.01	2.01	3.00	3.99	4.96
60	5	0.04	0.20	0.41	0.61	0.81	1.01
	10	0.08	0.38	0.76	1.13	1.51	1.88
	20	0.13	0.63	1.25	1.87	2.48	3.09
70	5	0.01	0.07	0.14	0.21	0.28	0.35
	10	0.06	0.28	0.55	0.83	1.10	1.38

For instance, we calculated the 20-year breast cancer risk for a 30-year old woman with a history of one abortion (code = 1), who was age 27 years at the first live birth (code = 1), no history of benign breast disease (code = 0), BMI of 27 (code = 1), with a family history of breast cancer (code = 1) and life satisfaction score of 7 (code = 0); the RR is derived as 2.512 × 1.895 × 1 × 1.372 × 3.250 × 1 = 21.23. Thus, in this example, the 20-year absolute risk would be 2.71% with RR of 20.0 (Table 3). The approximation was obtained as: 2.71 + (3.38–2.71) (21.23–20.00)/(25–20) = 2.87%, which means this woman has a 2.87% probability of developing breast cancer in the next 20 years. The approximation probability was close to the exact calculation of 2.88%.

### Evaluation of the HCBCP model in the Taixing prospective cohort study

In the Taixing Prospective Cohort Study, among 13,176 individuals who were breast cancer free at baseline, 34 had developed breast cancer after 7 years of follow-up. The incidence rate was 36.86/100,000 person-years.

**Fig. 2:**
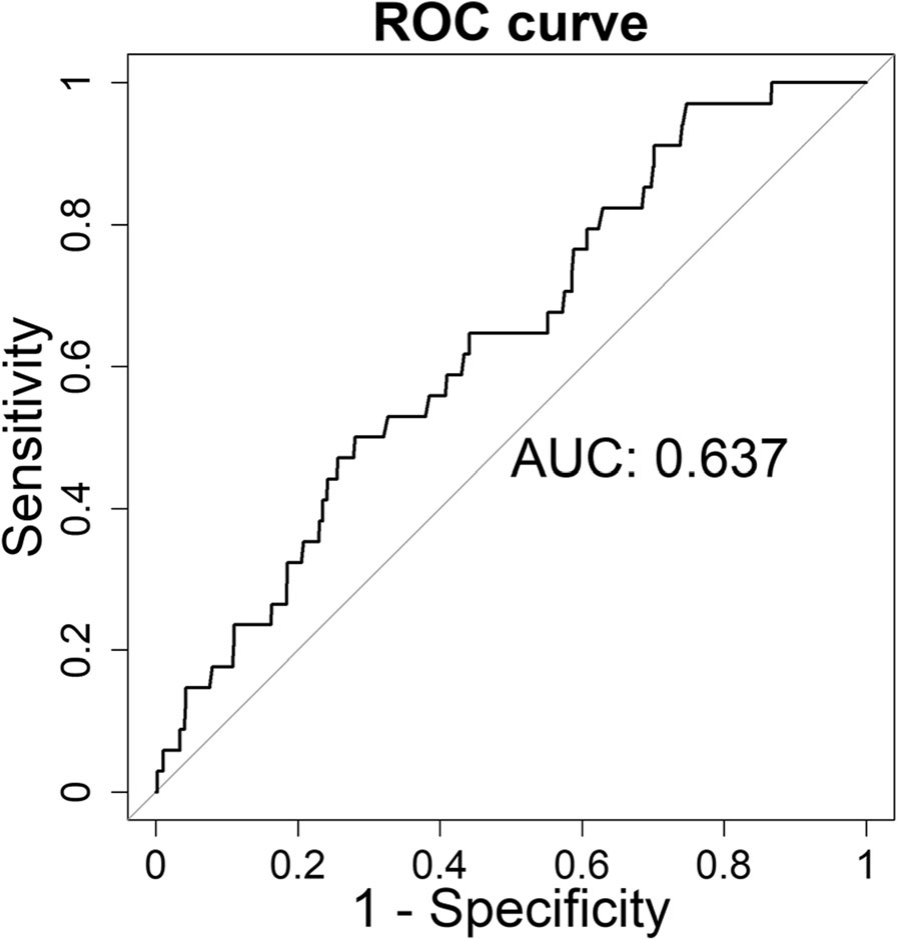
Discrimination performance of HCBCP model for breast cancer by C-statistic

The model calibration was assessed using the E/O ratio in the Taixing Prospective Cohort Study. The E/O ratio and 95% CI was 1.03 (0.74, 1.49). Receiver operating characteristic (ROC) curve analysis was performed to evaluate the discrimination ability of the HCBCP model. Figure 2 shows the results of ROC analysis to predict the absolute risk of breast cancer using the HCBCP model. The C-statistic was 0.64 (95% CI: 0.55, 0.72) with standard error 0.044.

### Comparison with other models

We compared the HCBCP model with the Gail model in the Taixing Prospective Cohort Study. Owing to a lack of information for the number of biopsies and biopsy atypical hyperplasia among study participants, these predictors were marked as unknown. The E/O ratio and 95% CI was 2.39 (1.71, 3.46), and the C-statistic was 0.54 (95% CI: 0.44, 0.63). The results showed that the Gail model tended to overestimate the absolute risks in the Taixing cohort. The Health risk appraisal (HRA) model [18], a risk assessment tool for breast cancer prediction among Chinese women, was also applied in the Taixing cohort; the E/O ratio was 1.88 (95% CI: 1.33, 2.75) and the C-statistic was 0.52 (95% CI: 0.43, 0.61).

## Discussion

We developed a risk prediction model for breast cancer for use in Han Chinese women. The results of validation showed good calibration and discriminative ability, with E/O ratio 1.03, and C-statistic 0.64 (95% CI: 0.55, 0.72). Using this model, women with a high risk of developing breast cancer can be identified using simple data collection. With the model, women identified as having high risk can be selected for further breast cancer-related examination, such as mammographic screening. Consequently, there is greater likelihood of identifying women with early-stage breast cancer. Women with higher risk might be motivated to maintain their current health status and take measures to prevent or delay the onset of breast cancer.

Competing risk is commonly existing in disease risk prediction. Traditional survival analysis models (like the Cox model) treat deaths from competing causes as independent censoring events. Hence, bias would be induced into risk prediction [19]. The proportional cause-specific hazard model is commonly used to analyze competing risks. The model computes absolute risk without assuming that these deaths act as “independent censoring” [19]. Therefore, we proposed a cause-specific hazard model to estimate absolute risk of an individual for the development of breast cancer.

Selection of the risk factors included in the current model was based on a systematic review of epidemiological studies as well as statistical analyses. In this study, we assessed a variety of factors that are considered to be associated with breast cancer including: number of abortions, age at first live birth, benign breast disease history, BMI, diabetes, breast cancer family history, and life satisfaction score; most risk factors have been confirmed in previous studies.

The relationship between diabetes and breast cancer is controversial. In our study, diabetes was a risk factor in univariate logistic regression (Additional file 2: Table S1) but it was not significant after multivariate-adjustment. This might be owing to the correlation between BMI and diabetes. Obesity is a major risk factor of diabetes. Weight-loss programs can lead to successful long-term weight loss and a decrease in the onset of diabetes [20, 21]. BMI has been found to be positively associated with increased risk of diabetes onset [22, 23]. Among women with gestational diabetes mellitus, BMI is significantly and positively associated with risk of progression from gestational diabetes mellitus to type 2 diabetes [24, 25]. However, in a meta-analysis, type 2 diabetes was found to increase the risk of breast cancer by 16% after adjustment for BMI [26]. One hypothesis is that hyperinsulinemia, as a potential risk factor of breast cancer and a marker of insulin resistance in obesity and type 2 diabetes, may account for the association among BMI, diabetes, and breast cancer [27–29]. Some studies have reported that increased BMI is associated with increased insulin [30]; therefore, BMI may be a confounding factor of diabetes and breast cancer. Compared with the model including diabetes, the model without diabetes showed a change in the E/O ratio from 0.99 to 1.03 as well as in the C-statistic, from 0.642 to 0.637; the 95% CI of the C-statistic also varied from 0.56–0.73 to 0.55–0.72), as did the standard error (from 0.043 to 0.044). Although the inclusion of diabetes in the final risk prediction model increased the C-statistic by 0.005, it was not significant in multivariate regression and the increase in discrimination was not distinct; thus, we did not include diabetes in the final model.

Life satisfaction scores have not been used in previous breast cancer prediction models. Many articles have reported that negative life events, depression, anxiety, and other harmful psychological and mental factors are related to breast cancer [31–33]. In our results, life satisfaction score showed a significance difference in both univariate and multivariate logistic regression. Life satisfaction status is a modifiable risk factor for which specific prevention measures can be implemented, which is important in community prevention of breast cancer.

Although additional nutrient variables including intakes of calcium, soy products, and iron have been related to breast cancer risk in some studies [34–36], a detailed dietary assessment and supporting nutritional database would be needed to accurately capture nutritional intake, making such assessment unfeasible in most clinical settings. Therefore, we did not include dietary variables that require a detailed assessment.

In China, some studies have added genetic markers to prediction models, to improve discriminative accuracy. Several prediction models have been developed that include some genetic markers and limited environmental predictors, with discriminative accuracy of around 0.6 [18, 37]. Together with the high cost of testing for genetic markers and the need to obtain blood samples, it is very difficult to implement such risk prediction tools among the general population of China.

In our developed model, all included risk factors are simple and feasible to measure, which improves its convenience and lowers the cost of implementation in a large population. In Western countries, the Gail model is used widely in clinic decision-making [38]. However, application of the Gail model in China is limited because biopsy tests are uncommon. Moreover, several validation studies have been conducted in Asian populations and have reported poor performance of the Gail model [39]. In a comparison with the Gail model in which race was defined as Chinese-American, the predicted incidence rate may still be higher than the actual rate in Taixing. Considering the lack of risk predictors and lower incidence rate in Taixing, Gail model’s prediction accuracy may be biased. In China, Yuan et al. developed a risk assessment tool for breast cancer prediction among Chinese women. Although that model’s C-statistic was 0.64 (95% CI: 0.50, 0.78), the authors included patients from a breast cancer database with data obtained in first-round screening only, with no further follow-up; these data may not be appropriate to test the reliability of the model [18]. In our study, the results of comparison showed that without including competing risks in the prediction model, the HRA model seemed to overestimate the probability of developing breast cancer in the Taixing cohort study. In our validation cohort, the E/O ratio was near 1 and showed good projection.

The strength of this study is we developed a simple and efficient prediction model with good calibration and discrimination. The E/O ratio was 1.03, which is very close to 1. The C-statistic was 0.64, which showed that the HCBCP model performed well in the validation study. Modifiable risk factors were selected, such that intervention measures can be carried out in high-risk groups. Using this model, populations at risk can easily be screened, to identify those with increased risk who would benefit from health management advice, to avoid or delay the onset of breast cancer.

There were also some limitations in our study. First, gene markers were not included in this model, which would lead to a decrease in the discriminative power. Second, we conducted the model using the age-specific breast cancer incidence rate and non-breast cancer mortality rate in Taixing; therefore, application of this HCBCP model may vary by geographic region. Third, the number of incident cases in the Taixing study was small (*n* = 34); therefore, the precision of the validation estimates may be affected. Fourth, owing to the administrative jurisdiction, the rate of loss to follow-up in the validation study was high and may bias the results. Hence, further studies in other areas of China are needed, to validate the performance of this model.

## Conclusions

In conclusion, we developed a risk prediction model including fertility status and relevant disease history as well as other modifiable risk factors. The developed model demonstrated good discriminative accuracy. We expect that the newly developed model can be used to screen populations with a high risk of breast cancer and will contribute to primary and secondary prevention of breast cancer in China.

## Additional files

Additional file 1: R code for HCBCP model. R code using to perform HCBCP model (DOCX 20 kb)

Additional file 2: **Table S1.** Relative Risk (RR) and 95% confidence interval (95% CI) of risk factors associated with breast cancer by univariate conditional logistic regression in Shandong Case Control Study (DOCX 15 kb)

Additional file 3: **Table S2.** The age-specific breast cancer incidence rates and non-breast cancer mortality rates of Taixing in 2015 (DOCX 14 kb)

Additional file 4: Questionnaire. An English language version of the questionnaire used in this study (PDF 84 kb)

## Abbreviations

AR: Attributable risk
BMI: Body mass index
HCBCP model: Han Chinese Breast Cancer Prediction model
ORs: Odds ratios
ROC: Receiver Operating Characteristic
RRs: Relative risks
