# Supplementary material for: Risk prediction for breast Cancer in Han Chinese women based on a cause-specific Hazard model
Source: BMC Cancer. 2019 Feb 7;19:128. doi: 10.1186/s12885-019-5321-1 (PMC6367757; doi:10.1186/s12885-019-5321-1)
Supplement: Supplementary file 1 — R code for HCBCP model. R code using to perform HCBCP model (DOCX 20 kb) [file 12885_2019_5321_MOESM1_ESM.docx]

**Supplementary Material——R code for HCBCP model**

Part 1、AR Calculate

setwd("E:/bcrisk/data")

#------compute rr

dd <- read.csv("test_nodia.csv",stringsAsFactors = F)#----import modeling data

r <- read.csv("logit_rr_nodia.csv",stringsAsFactors = F)#---- import beta of risk factors

dd1 <- dd[,which(colnames(dd)%in%r[,1])]

var <- r$Variable

rr <- rep(NA,nrow(dd1))

r1 <- rep(NA,length(var))

for (i in 1:nrow(dd1)){

for(j in 1:length(var)){

num <- dd1[i,which(colnames(dd1)%in%var[j])]

if (num==0) {r1[j]=1} else {

r1[j] <- exp(r[which(r$Variable==var[j]),2]*num)

}

}

rr[i] <- prod(r1)

}

RR_logit <- rr

res.rr <- data.frame(RR_logit,dd)

#-------compute Ft=1-AR

case <- res.rr[res.rr$case==1,]

Ft <- (1/nrow(case))*sum(1/case$RR_logit);Ft

Part 2、Cause-specific proportional model

setwd("E:/bcrisk/data")

#------compute rr

dd <- read.csv("test2_nodia.csv",stringsAsFactors = F) #----import validation data

dd <- dd[,-6]

r <- read.csv("logit_rr_nodia.csv",stringsAsFactors = F) #----import beta of risk factors

r[,1]<-c("bmi1","CancHist","FanHis","liuchan1","manyi1","shengyu1")

dd1 <- dd[,which(colnames(dd)%in%r[,1])]

var <- r$Variable

rr <- rep(NA,nrow(dd1))

r1 <- rep(NA,length(var))

for (i in 1:nrow(dd1)){

for(j in 1:length(var)){

num <- dd1[i,which(colnames(dd1)%in%var[j])]

if (num==0) {r1[j]=1} else {

r1[j] <- exp(r[which(r$Variable==var[j]),2]*num)

}

}

rr[i] <- prod(r1)

}

RR_logit <- rr

r_all <- data.frame(RR_logit,dd)

#-----------cause-specific model for absolute risk-------

RR <- r_all$RR_logit

age <- r_all$age

age2 <- r_all$age1

#incidence and mortality

# age-specific incident rate of breast cancer

h01 <- c(0.000000, 5.501163, 4.594786, 54.618354, 60.556036, 36.153430, 40.072531, 37.424379, 34.023442, 13.071383, 45.118646, 31.8203)/100000

# age-specific non-breast cancer mortality rate

h02 <- c(45.640000, 45.650000, 78.570782, 136.551226, 308.360416, 320.787925, 545.409120, 899.503612, 1265.385312, 1970.008585, 3639.187578,7191.3839) /100000

forerisk <- function (age1,age2,R,Ft){

SS1 <- NULL

SS2 <- NULL

age_zu <- seq(30,85,5) #

jmin <- match(T,age_zu > age1)

jmax <- match(T,age_zu > age2)

Delta <- rep(5,12)

hh1 <- h01 * Ft

hh2 <- h02

rr <- rep(R,12) #

if (jmax - jmin == 1){

delta <- c(age_zu[jmin] - age1, age2 - age_zu[jmin])

} else {

if(jmax - jmin == 0){

delta <- age2 - age1

} else {

delta <- c(age_zu[jmin] - age1, Delta[(jmin+1):(jmax-1)], age2 - age_zu[jmax-1])

}

}

h1 <- hh1[jmin:jmax]

h2 <- hh2[jmin:jmax]

r <- rr[jmin:jmax]

if(length(delta)>1){

for (i in 1:(length(delta)-1)){

SS1[i] <- exp(-sum(c( h1[1:i] * r[1:i] * delta[1:i])))

}

S1 <- c(1,SS1)

for (i in 1:(length(delta)-1)){

SS2[i] <- exp(-sum(c( h2[1:i] * delta[1:i])))

}

S2 <- c(1,SS2)

} else {

S1 <- 1

S2 <- 1

}

P <- h1 * r / (h1 * r + h2) * S1 * S2 * (rep(1,length(h1)) - 1/exp(delta * (h1 * r + h2)))

sum(P)

}

cohortrisk<- NULL

for(i in 1:nrow(r_all)){

cohortrisk[i] <- forerisk(age1=age[i],age2=age2[i],R=RR[i],Ft) }

result <- data.frame(r_all,cohortrisk)
