# Supplementary material for: Risk prediction for breast Cancer in Han Chinese women based on a cause-specific Hazard model
Source: BMC Cancer. 2019 Feb 7;19:128. doi: 10.1186/s12885-019-5321-1 (PMC6367757; doi:10.1186/s12885-019-5321-1)
Supplement: Supplementary file 2 — Table S1. Relative Risk (RR) and 95% confidence interval (95% CI) of risk factors associated with breast cancer by univariate conditional logistic regression in Shandong Case Control Study (DOCX 15 kb) [file 12885_2019_5321_MOESM2_ESM.docx]

**Supplementary Table S1.** Relative Risk (RR) and 95% confidence interval (95%CI) of risk factors associated with breast cancer by univariate conditional logistic regression in Shandong Case Control Study

| Variable | | Code | RR | 95% *CI* | | *P*-value |
| --- | --- | --- | --- | --- | --- | --- |
| Number of abortions | 0 | 0 | 1.000 | - | | <0.001 |
|  | 1-2 | 1 | 2.823 | 2.190 | 3.638 |  |
|  | ≥3 | 2 | 7.966 | 6.180 | 10.268 |  |
| Age at first live birth | <25 | 0 | 1.000 | - | | <0.001 |
|  | 25-29 | 1 | 1.971 | 1.553 | 2.502 |  |
|  | ≥30 | 2 | 3.885 | 3.060 | 4.931 |  |
| Benign breast disease history | No | 0 | 1.000 | - | | <0.001 |
|  | Yes | 1 | 5.143 | 2.148 | 12.313 |  |
| BMI | <24 | 0 | 1.000 | - | | 0.002 |
|  | 24-27.9 | 1 | 1.375 | 1.128 | 1.675 |  |
|  | ≥28 | 2 | 1.890 | 1.551 | 2.303 |  |
| Diabetes | No | 0 | 1.000 | - | | 0.017 |
|  | Yes | 1 | 2.318 | 1.164 | 4.614 |  |
| Breast cancer family history | No | 0 | 1.000 | - | | 0.000 |
|  | Yes | 1 | 4.222 | 1.910 | 9.332 |  |
| Life satisfaction scores | <13 | 0 | 1.000 | - | | <0.001 |
|  | ≥13 | 1 | 2.555 | 1.927 | 3.387 |  |
