# Supplementary material for: Risk prediction for breast Cancer in Han Chinese women based on a cause-specific Hazard model
Source: BMC Cancer. 2019 Feb 7;19:128. doi: 10.1186/s12885-019-5321-1 (PMC6367757; doi:10.1186/s12885-019-5321-1)
Supplement: Supplementary file 3 — Table S2. The age-specific breast cancer incidence rates and non-breast cancer mortality rates of Taixing in 2015 (DOCX 14 kb) [file 12885_2019_5321_MOESM3_ESM.docx]

**Supplementary Table S2.** The age-specific breast cancer incidence rates and non-breast cancer mortality rates of Taixing in 2015

| Age group | Incidence rate（/100,000） | Non-breast cancer mortality rate（/100,000） |
| --- | --- | --- |
| <20 | 0.0000 | 19.9900 |
| 20- | 0.0000 | 27.5300 |
| 25- | 0.0000 | 45.6400 |
| 30- | 5.5012 | 45.6500 |
| 35- | 4.5948 | 78.5708 |
| 40- | 54.6184 | 136.5512 |
| 45- | 60.5560 | 308.3604 |
| 50- | 36.1534 | 320.7879 |
| 55- | 40.0725 | 545.4091 |
| 60- | 37.4244 | 899.5036 |
| 65- | 34.0234 | 1265.3853 |
| 70- | 13.0714 | 1970.0086 |
| 75- | 45.1186 | 3639.1876 |
| 80- | 31.8203 | 7191.3839 |
| 85 | 41.2312 | 28945.3860 |
