# Supplementary material for: Risk prediction for breast Cancer in Han Chinese women based on a cause-specific Hazard model
Source: BMC Cancer. 2019 Feb 7;19:128. doi: 10.1186/s12885-019-5321-1 (PMC6367757; doi:10.1186/s12885-019-5321-1)
Supplement: Supplementary file 4 — Questionnaire. An English language version of the questionnaire used in this study (PDF 84 kb) [file 12885_2019_5321_MOESM4_ESM.pdf]

Questionnaire of women health

No 1________

No 2 ________

I. Basic information

1. Name____________, Location______

Birthdate：______, Age______, Telephone_________________

1.1 Living environment (1)Urban (2)Suburban (3)Rural

1.2 Registered residence (1)Urban (2))Rural

2. Occupation:(1)Farmer,(2)Worker,(3)Teacher, (4)Civil service, (5)Individual traders,(6)Driver,(7)Services,(8)Staff,(9)Housewife,（10）Health care,（11）Student （12）Other________

3.Education level:_________ Education Year

3.1 Highest level of education: (1)Primary or below (2)Junior high school (3)Senior middle or vocational high school (4)University (5)Post-graduate

4. Height____meters (with two decimal points 1.62); Weight____Kg(with one decimal points, 52.5)

4.1 Waistline meters, Hipcircumference meters

5. Number of family members ,Personal annual income ,Family annual income

5.1 How is your family economic status？ 1=Very good 2=Good 3=Common 4=Poor 5=Very poor

5.2 How is your family social status？ 1=Very good 2=Good 3=Common 4=Poor 5=Very poor

6 Marital status

6.1Marital status:(1)Single; (2)Married and living together; (3)Married but separated >3 months; (4)Divorced; (5) Windowed （6）Married after divorce （7）Unmarried cohabitation （8）Other ____________

6.2 Parity____ ; Pregnancy______; Miscarriages______, if yes, age at 1st miscarriage______

6.3 Times of full term produce _____ 88=not suitable

6.4 Age at 1st pregnancy at term____ 88= not suitable

6.5Children？ _______ 88= not suitable

6.5.1 Boys______个, 88= not suitable

6.5.2 Girls ____个, 88= not suitable

6.5.3 Full term?:(1)Yes , (2)No 88= not suitable

6.5.4 Breast feeding？ (1)Yes, (2)No; 88= not suitable

6.5.5 If yes ,breastfeeding duration________months 88= not suitable

6.6 Use of contraceptive drugs? (1)Yes (2) No 88= not suitable

6.6.1 If yes, use duration______years 88= not suitable

7. Physiological state

7.1 Age at menarche_____; Menstrual pattern, regular？ (1)Yes, (2)No

7.2 Menstrual period____days , Intermenstrual period_____days

7.3 Dysmenorrhea: (1)Yes, (2)No;

7.4 Postmenopausal？ (1)Yes, (2)No; If yes, age at menopause___

If yes, use of hormone replacement (1)Yes , (2)No ; 88=not suitable

If postmenopausal, weight gain? (1)Yes , (2)No, If yes, how many________

II. Disease and family history

(1) Disease history

8.1 Ever diagnosed as breast cancer? If yes, when yy/mm, where A/B/C degree hospital

8.2 Ever diagnosed as benign breast tumor? If yes, when yy/mm, where A/B/C degree hospital

8.3 Ever diagnosed as breast hyperplasia? If yes, when yy/mm, where A/B/C degree hospital

8.4 Ever diagnosed as nipple discharge? If yes, when yy/mm, where A/B/C degree hospital

8.5 Ever diagnosed as accessory breast? If yes, when yy/mm, where A/B/C degree hospital

8.6 Ever diagnosed as retracted nipple? If yes, when yy/mm, where A/B/C degree hospital

8.7 Ever diagnosed as other gynecological neoplasms ?

8.7.1 Cervical cancer: (1)Yes,(2) No; If yes, when yy/mm, where A/B/C degree hospital

8.7.2 Ovarian cancer: (1)Yes,(2) No; If yes, when yy/mm, where A/B/C degree hospital

8.7.3 Ovarian cyst: (1)Yes, (2) No; If yes, when yy/mm, where A/B/C degree hospital

8.8 Ever diagnosed as other chronic disease?

8.8.1 Diabetes mellitus: (1)Yes, (2) No; If yes, when yy/mm, where A/B/C degree hospital

8.8.2 Hypertension: (1)Yes, (2) No; If yes, when yy/mm, where A/B/C degree hospital

8.8.3 Coronary heart disease: (1)Yes, (2) No; If yes, when yy/mm, where A/B/C degree hospital

8.8.4 Other malignancies: (1)Yes, (2) No; If yes, when yy/mm, where A/B/C degree hospital

8.8.5 Nephritis: (1)Yes, (2) No; If yes, when yy/mm, where A/B/C degree hospital

8.8.6 Mental disorders: (1)Yes, (2) No; If yes, when yy/mm, where A/B/C degree hospital

(2) Breast cancer family history

9.1 First-degree relatives (father, mother, son, daughter, brothers and sisters) ever diagnosed as breast cancer?

(1)Yes ,(2)No ; If yes, Who________________, How many____________

9.2 Second-degree relatives (uncle, aunt, grandparents,) ever diagnosed as breast cancer?

(1)Yes ,(2)No ; If yes, Who________________, How many____________

9.3 Third-degree relatives (cousins) ever diagnosed as breast cancer?

(1)Yes ,(2)No ; If yes, Who________________, How many____________

III. Living habits

10 Smoking

10.1 Do you smoke? (1)Never (2)Occationally （3）often

10.2 Age at first smoking _____, How many years of smoking______(Duration excluded, when quit smoking)。

10.3 When smoking, how many cigarretes per day______

10.4 If you don’t smoke, Do people around smoke? (1)Yes, (2)No

11 Drinking

11.1 Do you drink？ (1)Never (2)Occationally （3）often

11.2 If yes, how many years

11.3 Age at first drink？

11.4 Have you got drunk？ (1)Never(2)1-2 times(3) 3-5 times(4) 6-9 times(5)10times or over

11.5 Type of drinking (1) white spirit;(2)beer;(3)red wine;(4)white wine (5)other

11.6 Average drinking volume?_________,how many times do you drink every month? _____

12 Diet: 1=Nearly everyday（5-7d every week）, 2= 3-4d every week, 3= 1-2d every week, 4=almost never

12.1 Bean product（bean curd, bean milk）	1	2	3	4
12.2 Fresh beans（green soyabeans, broad bean）	1	2	3	4
12.3 Red meat（pork, mutton, beef）	1	2	3	4
12.4 Dairy products:	1	2	3	4

12.5 Corn products	1	2	3	4
12.6 Carrot	1	2	3	4
12.7 Fried foods（fried meat, fried pasta）	1	2	3	4
12.8 Colored vegetables or fruit	1	2	3	4
12.9 Garlic	1	2	3	4
12.10 Ham	1	2	3	4
12.11 Pickled food（brined vegetable, salted egg, salted meat）	1	2	3	4

13.Tea? (1)Yes (2)No If yes, what kind of tea (1)green tea (2)black tea (3)other____________

14 Coffee? (1)Yes (2)No

15 Sleep

15.1 How many hours do you sleep? Since when yy mm

15.2 Always have trouble sleeping? (1)Yes (2)No

15.3 Always have nightmares? (1)Yes, (2)No Since when yy mm

15.4 Do you have early-wake? (1)Yes (2)No Since when yy mm

15.5 Do you ever sleep late? (1)Yes (2)No Since when yy mm

15.6 Are you satisfied with your sleep? ①very satisfied ②satisfied ③dissatisfied ④very dissatisfied

16 Sports activites

16.1 Do you do exercises? (1)Never (2)Occasionally (3)Often

16.2 How often do you do exercises? (1)< 3 times every week (2)⩾ 3 times every week

16.3 Your Current amount of exercise? (1) < 30 minutes every day (2) ⩾ 30 minutes every day

17. Personality

17.1 Are you introvert? (1) Yes (2)Ordinary (3)No

17.2 Are you full of passion? (1) Yes (2)Ordinary (3)No

17.3 Are you impatient? (1) Yes (2)Ordinary (3)No

18 Life satisfactory degree 1=Very satisfied 2=Satisfied 3=Common 4=Dissatisfied 5=Very dissatisfied 88=Not suitable

18.1 **Current** life	1	2	3	4	5	88
18.2 **Current** income	1	2	3	4	5	**88**
18. **Current** physical status	1	2	3	4	5	88
18.4 **Current** housing conditions:	1	2	3	4	5	88
18.5 **Current** marital status	1	2	3	4	5	**88**
18.6 **Current** medical conditions	1	2	3	4	5	88
18.7 **Current** neighborhood	1	2	3	4	5	88
18.8 Life of **six months ago**	1	2	3	4	5	88
18.9 Life of **1 year ago**	1	2	3	4	5	88
18.10 Life of **5 years ago**	1	2	3	4	5	88
18.11 Life of **1 year later**	1	2	3	4	5	88
18.12 Life of **5 years later**	1	2	3	4	5	88

IV. Medications or use of chemical drugs

19.1 Antidepressant drug (1)No (2)Yes; If yes, Since when yy mm

19.2 Antihistamine drug (1)No (2)Yes; If yes, Since when yy mm

19.3 Diuretic (1)No (2)Yes; If yes, Since when yy mm

19.4 Antiemetic drugs (1)No (2)Yes; If yes, Since when yy mm

19.5 Antihypertensive drugs (1)No (2)Yes; If yes, Since when yy mm

19.6 Sleeping pills (1)No (2)Yes; If yes, Since when yy mm
19.7 Estrogenic drug: (1)No (2)Yes; If yes, Since when yy mm
19.8 Hair dye (1)No (2)Yes; If yes, Since when yy mm

V. Other

20.1 Do you usually concern for breast care? (1)Never (2) Occationally（3）often

20.2 Do you know breast self-examine? (1)Yes (2)No

20.3 Have you ever examined your own breast? (1)Yes (2)No

20.4 Do you regularly go to special hospital for breast examinations ? (1)Yes (2)No

20.5Do you know breast cancer? (1)Yes (2)No

20.6Do you think screening is helpful for early detection of breast cancer? (1)Yes (2)No

20.7Do you think the early detection of breast cancer can improve survival? (1)Yes (2)No

20.8 Have you ever done breast screening? (1)Yes (2)No

a. Have you ever received the clinical touch (1)Yes (2)No

b. Have you ever done X-ray examination? (1)Yes (2)No

c. Have you ever done breast ultrasound？ (1)Yes (2)No

20.9 knowledge about breast symptoms

a. Do you think the local discomfort of breast is a signal of breast cancer occurrence?

(1)Yes (2) No (3)Don’t know

b. Do you think the lump in your breast is a signal of breast cancer occurrence ?

(1)Yes (2) No (3)Don’t know

c. Do you think the axillary nodes is a signal of breast cancer occurrence ?

(1)Yes (2) No (3)Don’t know

d. Do you think the nipple retraction is a signal of breast cancer occurrence ?

(1)Yes (2) No (3)Don’t know

e.Do you think the nipple discharge liquid is a signal of breast cancer occurrence ?

(1)Yes (2) No (3)Don’t know

20.10 Related factors of breast cancer

a.Do you think age at menarche before 12 may cause breast cancer?

(1)Yes (2) No (3)Don’t know

b.Do you think no parityor late childbirth may cause breast cancer?

(1)Yes (2) No (3)Don’t know

c. Do you think menopause at a late age may cause breast cancer?

(1)Yes (2) No (3)Don’t know

d. Do you think long time drinking may cause breast cancer?

(1)Yes (2) No (3)Don’t know

e. Do you think high-fat diets may cause breast cancer?

(1)Yes (2) No (3)Don’t know

f. Do you think Long-term use of estrogen drugs may cause breast cancer?

(1)Yes (2) No (3)Don’t know

g. Do you think people who have a family history of breast cancer is more likely to have breast cancer? (1)Yes (2) No (3)Don’t know
